# Supplementary material for: Daylight Saving Time and Acute Myocardial Infarction: A Meta-Analysis
Source: J Clin Med. 2019 Mar 23;8(3):404. doi: 10.3390/jcm8030404 (PMC6463000; doi:10.3390/jcm8030404)
Supplement: Supplementary file 1 [file jcm-08-00404-s001.pdf]

Daylight saving time and acute myocardial infarction: a meta-analysis.

Supplementary material

Table S1. List of the excluded studies, and reasons for the exclusion.

First author - Year	Reason for the exclusion
Foerch 2008 [1]	Only data on pre-post- DST stroke incidence reported
Sipilä 2016 [2]	Only data on pre-post- DST stroke incidence reported
Lindenberger 2018 [3]	Only data on forensic autopsies in the week following DST reported (absence of pre- post-DST comparisons)
Manfredini 2018 [4]	No additional data provided (review of previously published studies)

Figure S1. Risk of acute myocardial infarction (AMI) during the first week following daylight saving time (DST) transitions versus control weeks – **Overall**.

Study or Subgroup	log[Odds Ratio]	SE	Weight	Odds Ratio IV, Random, 95% CI	Year
Janszky 2008s	0.0488	0.0098	13.6%	1.05 [1.03, 1.07]	2008
Janszky 2008a	-0.0101	0.0104	13.4%	0.99 [0.97, 1.01]	2008
Janszky 2012s	0.0392	0.02	10.8%	1.04 [1.00, 1.08]	2012
Janszky 2012a	0	0.0155	12.1%	1.00 [0.97, 1.03]	2012
Culic 2013s	0.1398	0.0513	4.3%	1.15 [1.04, 1.27]	2013
Jiddou 2013s	0.157	0.0801	2.1%	1.17 [1.00, 1.37]	2013
Jiddou 2013a	-0.0101	0.0778	2.2%	0.99 [0.85, 1.15]	2013
Culic 2013a	0.174	0.0542	3.9%	1.19 [1.07, 1.32]	2013
Sandhu 2014a	-0.0202	0.0434	5.3%	0.98 [0.90, 1.07]	2014
Sandhu 2014s	0.0296	0.0413	5.7%	1.03 [0.95, 1.12]	2014
Kirchberger 2015a	0.0296	0.0521	4.2%	1.03 [0.93, 1.14]	2015
Kirchberger 2015s	0.077	0.0496	4.5%	1.08 [0.98, 1.19]	2015
Sipila 2015s	0.01	0.0259	9.1%	1.01 [0.96, 1.06]	2015
Sipila 2015a	-0.0101	0.0264	8.9%	0.99 [0.94, 1.04]	2015
**Total (95% CI)**			**100.0%**	**1.03 [1.01, 1.06]**	

Heterogeneity: Tau² = 0.00; Chi² = 39.83, df = 13 (P = 0.0001); I² = 67%

Test for overall effect: Z = 2.44 (P = 0.01)

Odds Ratio
IV, Random, 95% CI

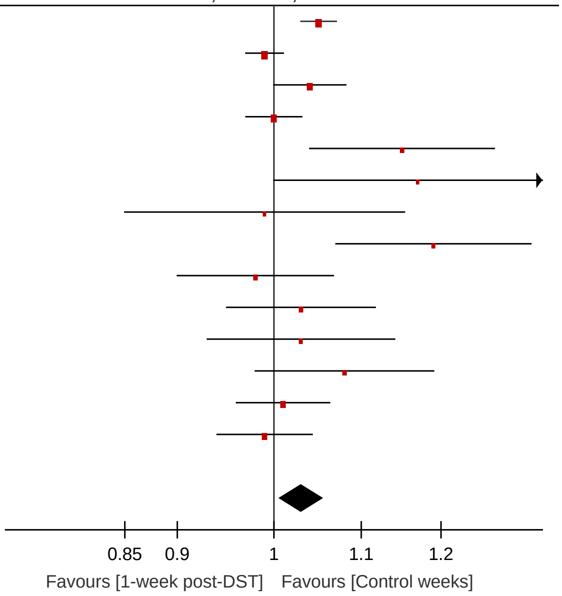

In all studies, "a" = "autumn DST transition", "s" = "spring DST transition".

Figure S2. Risk of acute myocardial infarction (AMI) during the first week following daylight saving time (DST) transition versus control weeks – **Females only**.

Study or Subgroup	log[Odds Ratio]	SE	Weight	Odds Ratio IV, Random, 95% CI	Year
Janszky 2012a	-0.0101	0.0264	40.4%	0.99 [0.94, 1.04]	2012
Janszky 2012s	0.0677	0.0294	38.1%	1.07 [1.01, 1.13]	2012
Culic 2013s	-0.5447	0.3364	1.0%	0.58 [0.30, 1.12]	2013
Culic 2013a	0.4383	0.3311	1.0%	1.55 [0.81, 2.97]	2013
Kirchberger 2015s	0	0.0951	10.3%	1.00 [0.83, 1.20]	2015
Kirchberger 2015a	-0.0101	0.1024	9.1%	0.99 [0.81, 1.21]	2015
**Total (95% CI)**			**100.0%**	**1.02 [0.95, 1.09]**	

Heterogeneity: Tau² = 0.00; Chi² = 8.47, df = 5 (P = 0.13); I² = 41%

Test for overall effect: Z = 0.59 (P = 0.56)

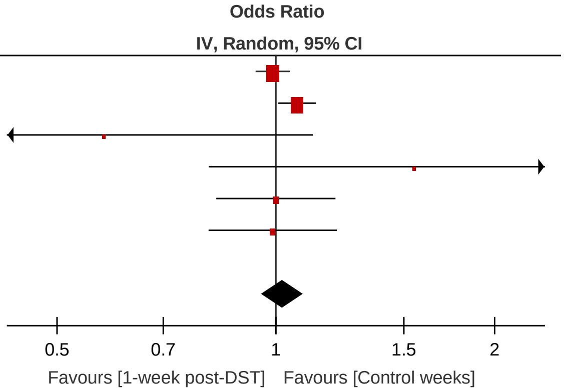

In all studies, "a" = "autumn DST transition", "s" = "spring DST transition".

Figure S3. Risk of acute myocardial infarction (AMI) during the first week following daylight saving time (DST) transition versus control weeks – **Males only**.

Study or Subgroup	log[Odds Ratio]	SE	Weight	Odds Ratio IV, Random, 95% CI	Year
Janszky 2012a	0	0.0208	33.1%	1.00 [0.96, 1.04]	2012
Janszky 2012s	0.0198	0.0204	33.7%	1.02 [0.98, 1.06]	2012
Culic 2013a	-0.4308	0.3306	0.3%	0.65 [0.34, 1.24]	2013
Culic 2013s	0.9895	0.4849	0.1%	2.69 [1.04, 6.96]	2013
Kirchberger 2015a	0.0392	0.0626	7.7%	1.04 [0.92, 1.18]	2015
Kirchberger 2015s	0.1044	0.0584	8.6%	1.11 [0.99, 1.24]	2015
Sipila 2015a	-0.0101	0.0543	9.7%	0.99 [0.89, 1.10]	2015
Sipila 2015s	0.0488	0.0674	6.7%	1.05 [0.92, 1.20]	2015
**Total (95% CI)**			**100.0%**	**1.02 [0.98, 1.06]**	

Heterogeneity: Tau² = 0.00; Chi² = 9.38, df = 7 (P = 0.23); I² = 25%

Test for overall effect: Z = 1.13 (P = 0.26)

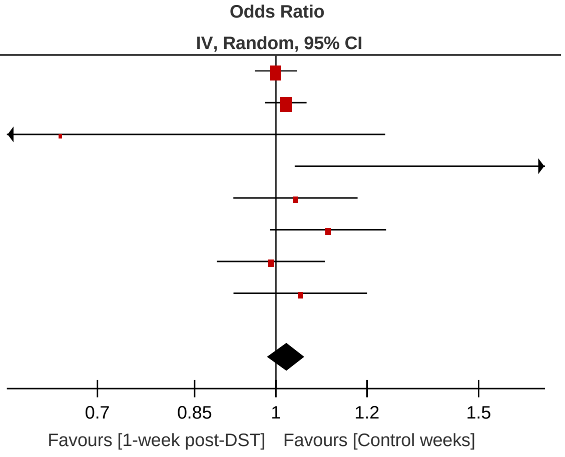

In all studies, "a" = "autumn DST transition", "s" = "spring DST transition".

Figure S4. Risk of acute myocardial infarction (AMI) during the first week following daylight saving time (DST) transition versus control weeks – **Age <65 years only**.

Study or Subgroup	log[Odds Ratio]	SE	Weight	Odds Ratio IV, Random, 95% CI	Year
Janszky 2012a	0	0.0262	50.5%	1.00 [0.95, 1.05]	2012
Janszky 2012s	0.01	0.0312	35.6%	1.01 [0.95, 1.07]	2012
Kirchberger 2015s	0.0198	0.0696	7.2%	1.02 [0.89, 1.17]	2015
Kirchberger 2015a	0.0677	0.0715	6.8%	1.07 [0.93, 1.23]	2015
**Total (95% CI)**			**100.0%**	**1.01 [0.97, 1.05]**	

Heterogeneity: Tau² = 0.00; Chi² = 0.82, df = 3 (P = 0.85); I² = 0%

Test for overall effect: Z = 0.51 (P = 0.61)

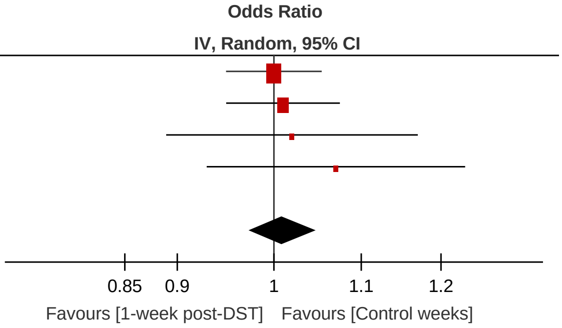

In all studies, "a" = "autumn DST transition", "s" = "spring DST transition".

Figure S5. Risk of acute myocardial infarction (AMI) during the first week following daylight saving time (DST) transition versus control weeks – **Age ≥65 years only**.

Study or Subgroup	log[Odds Ratio]	SE	Weight	Odds Ratio IV, Random, 95% CI	Year
Janszky 2012a	-0.0101	0.0157	42.0%	0.99 [0.96, 1.02]	2012
Janszky 2012s	0.0488	0.0198	38.7%	1.05 [1.01, 1.09]	2012
Kirchberger 2015s	0.1398	0.0764	9.9%	1.15 [0.99, 1.34]	2015
Kirchberger 2015a	-0.0202	0.0786	9.4%	0.98 [0.84, 1.14]	2015
**Total (95% CI)**			**100.0%**	**1.03 [0.97, 1.08]**	

Heterogeneity: Tau² = 0.00; Chi² = 8.34, df = 3 (P = 0.04); I² = 64%

Test for overall effect: Z = 0.99 (P = 0.32)

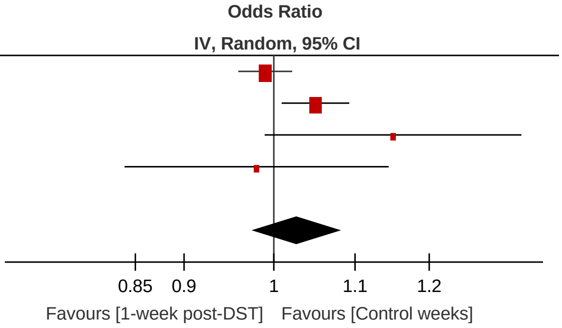

In all studies, "a" = "autumn DST transition", "s" = "spring DST transition".

Figure S6. Risk of acute myocardial infarction (AMI) during the first week following daylight saving time (DST) spring transition versus control weeks – **Overall**.

Study or Subgroup	log[Odds Ratio]	SE	Weight	Odds Ratio IV, Random, 95% CI	Year
Janszky 2008s	0.0488	0.0098	43.1%	1.05 [1.03, 1.07]	2008
Janszky 2012s	0.0392	0.02	22.2%	1.04 [1.00, 1.08]	2012
Culic 2013s	0.1398	0.0513	4.9%	1.15 [1.04, 1.27]	2013
Jiddou 2013s	0.157	0.0801	2.1%	1.17 [1.00, 1.37]	2013
Sandhu 2014s	0.0296	0.0413	7.2%	1.03 [0.95, 1.12]	2014
Kirchberger 2015s	0.077	0.0496	5.2%	1.08 [0.98, 1.19]	2015
Sipila 2015s	0.01	0.0259	15.5%	1.01 [0.96, 1.06]	2015
**Total (95% CI)**			**100.0%**	**1.05 [1.02, 1.07]**	

Heterogeneity: Tau² = 0.00; Chi² = 7.93, df = 6 (P = 0.24); I² = 24%

Test for overall effect: Z = 4.03 (P < 0.0001)

Odds Ratio
IV, Random, 95% CI

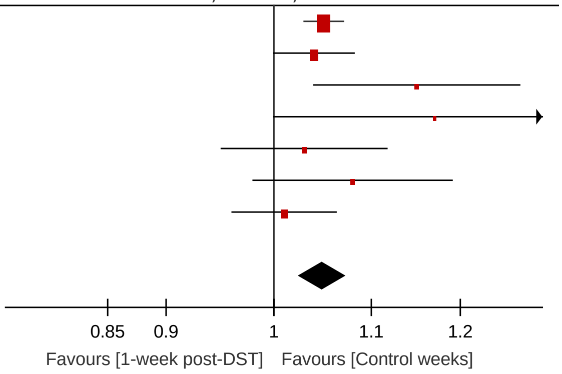

In all studies, "a" = "autumn DST transition", "s" = "spring DST transition".

Figure S7. Risk of acute myocardial infarction (AMI) during the first week following daylight saving time (DST) <u>spring</u> transition versus control weeks – **<u>Females only</u>**.

Study or Subgroup	log[Odds Ratio]	SE	Weight	Odds Ratio IV, Random, 95% CI	Year
Janszky 2012s	0.0677	0.0294	63.1%	1.07 [1.01, 1.13]	2012
Culic 2013s	-0.5447	0.3364	4.5%	0.58 [0.30, 1.12]	2013
Kirchberger 2015s	0	0.0951	32.4%	1.00 [0.83, 1.20]	2015
**Total (95% CI)**			**100.0%**	**1.02 [0.88, 1.18]**	

Heterogeneity: $Tau^2 = 0.01$; $Chi^2 = 3.69$, df = 2 (P = 0.16); $I^2 = 46\%$

Test for overall effect: Z = 0.25 (P = 0.80)

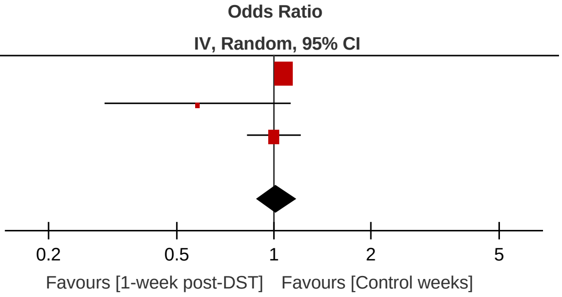

In all studies, "a" = "autumn DST transition", "s" = "spring DST transition".

Figure S8. Risk of acute myocardial infarction (AMI) during the first week following daylight saving time (DST) spring transition versus control weeks – **Males only**.

Study or Subgroup	log[Odds Ratio]	SE	Weight	Odds Ratio IV, Random, 95% CI	Year
Janszky 2012s	0.0198	0.0204	49.3%	1.02 [0.98, 1.06]	2012
Culic 2013s	0.9895	0.4849	0.7%	2.69 [1.04, 6.96]	2013
Sipila 2015s	0.0488	0.0674	23.0%	1.05 [0.92, 1.20]	2015
Kirchberger 2015s	0.1044	0.0584	26.9%	1.11 [0.99, 1.24]	2015
**Total (95% CI)**			**100.0%**	**1.06 [0.97, 1.15]**	

Heterogeneity: Tau² = 0.00; Chi² = 5.86, df = 3 (P = 0.12); I² = 49%

Test for overall effect: Z = 1.34 (P = 0.18)

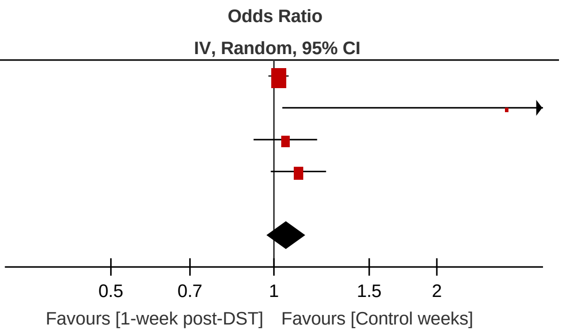

In all studies, "a" = "autumn DST transition", "s" = "spring DST transition".

Figure S9. Risk of acute myocardial infarction (AMI) during the first week following daylight saving time (DST) spring transition versus control weeks – **Age <65 years only**.

Study or Subgroup	log[Odds Ratio]	SE	Weight	Odds Ratio IV, Random, 95% CI	Year	Odds Ratio IV, Random, 95% CI
Janszky 2012s	0.01	0.0312	83.3%	1.01 [0.95, 1.07]	2012	
Kirchberger 2015s	0.0198	0.0696	16.7%	1.02 [0.89, 1.17]	2015	
**Total (95% CI)**			**100.0%**	**1.01 [0.96, 1.07]**		

Heterogeneity: Tau² = 0.00; Chi² = 0.02, df = 1 (P = 0.90); I² = 0%

Test for overall effect: Z = 0.41 (P = 0.68)

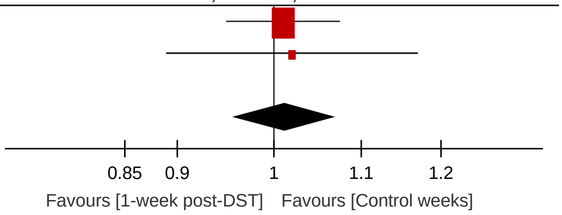

In all studies, "a" = "autumn DST transition", "s" = "spring DST transition".

Figure S10. Risk of acute myocardial infarction (AMI) during the first week following daylight saving time (DST) spring transition versus control weeks – **Age ≥65 years only**.

Study or Subgroup	log[Odds Ratio]	SE	Weight	Odds Ratio IV, Random, 95% CI	Year
Janszky 2012s	0.0488	0.0198	82.9%	1.05 [1.01, 1.09]	2012
Kirchberger 2015s	0.1398	0.0764	17.1%	1.15 [0.99, 1.34]	2015
**Total (95% CI)**			**100.0%**	**1.07 [1.00, 1.14]**	

Heterogeneity: Tau² = 0.00; Chi² = 1.33, df = 1 (P = 0.25); I² = 25%

Test for overall effect: Z = 1.88 (P = 0.06)

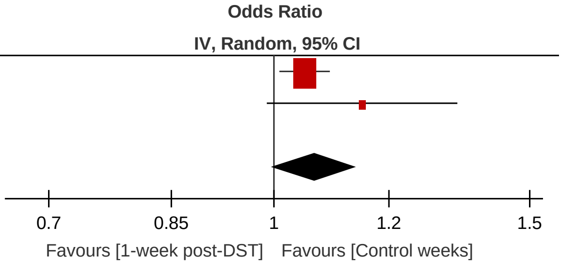

In all studies, "a" = "autumn DST transition", "s" = "spring DST transition".

Figure S11. Risk of acute myocardial infarction (AMI) during the first week following daylight saving time (DST) autumn transition versus control weeks – **Overall**.

Study or Subgroup	log[Odds Ratio]	SE	Weight	Odds Ratio IV, Random, 95% CI	Year
Janszky 2008a	-0.0101	0.0104	31.4%	0.99 [0.97, 1.01]	2008
Janszky 2012a	0	0.0155	26.4%	1.00 [0.97, 1.03]	2012
Culic 2013a	0.174	0.0542	6.2%	1.19 [1.07, 1.32]	2013
Jiddou 2013a	-0.0101	0.0778	3.3%	0.99 [0.85, 1.15]	2013
Sandhu 2014a	-0.0202	0.0434	8.9%	0.98 [0.90, 1.07]	2014
Kirchberger 2015a	0.0296	0.0521	6.7%	1.03 [0.93, 1.14]	2015
Sipila 2015a	-0.0101	0.0264	17.1%	0.99 [0.94, 1.04]	2015
**Total (95% CI)**			**100.0%**	**1.01 [0.98, 1.04]**	

Heterogeneity: Tau² = 0.00; Chi² = 11.80, df = 6 (P = 0.07); I² = 49%

Test for overall effect: Z = 0.39 (P = 0.70)

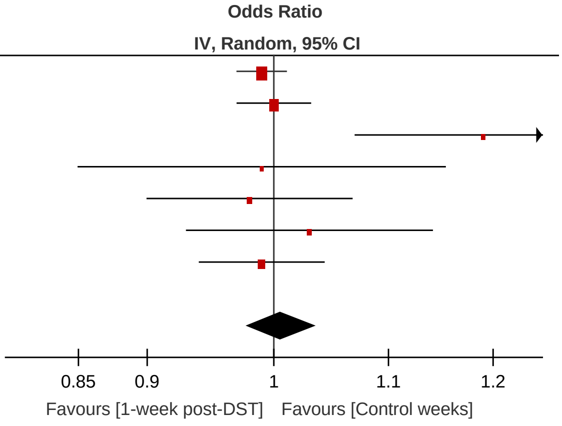

In all studies, "a" = "autumn DST transition", "s" = "spring DST transition".

Figure S12. Risk of acute myocardial infarction (AMI) during the first week following daylight saving time (DST) autumn transition versus control weeks – **Females only**.

Study or Subgroup	log[Odds Ratio]	SE	Weight	Odds Ratio IV, Random, 95% CI	Year
Janszky 2012a	-0.0101	0.0264	93.2%	0.99 [0.94, 1.04]	2012
Culic 2013a	0.4383	0.3311	0.6%	1.55 [0.81, 2.97]	2013
Kirchberger 2015a	-0.0101	0.1024	6.2%	0.99 [0.81, 1.21]	2015
**Total (95% CI)**			**100.0%**	**0.99 [0.94, 1.04]**	

Heterogeneity: Tau² = 0.00; Chi² = 1.82, df = 2 (P = 0.40); I² = 0%

Test for overall effect: Z = 0.29 (P = 0.77)

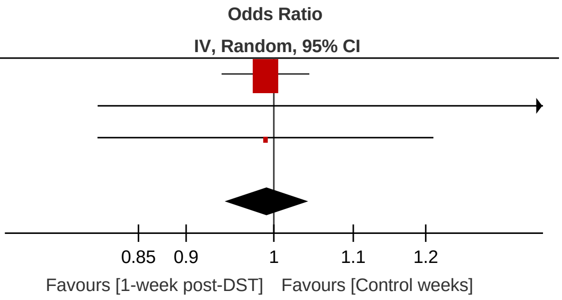

In all studies, "a" = "autumn DST transition", "s" = "spring DST transition".

Figure S13. Risk of acute myocardial infarction (AMI) during the first week following daylight saving time (DST) autumn transition versus control weeks – **Males only**.

Study or Subgroup	log[Odds Ratio]	SE	Weight	Odds Ratio IV, Random, 95% CI	Year
Janszky 2012a	0	0.0208	79.3%	1.00 [0.96, 1.04]	2012
Culic 2013a	-0.4308	0.3306	0.3%	0.65 [0.34, 1.24]	2013
Sipila 2015a	-0.0101	0.0543	11.6%	0.99 [0.89, 1.10]	2015
Kirchberger 2015a	0.0392	0.0626	8.8%	1.04 [0.92, 1.18]	2015
**Total (95% CI)**			**100.0%**	**1.00 [0.97, 1.04]**	

Heterogeneity: Tau² = 0.00; Chi² = 2.12, df = 3 (P = 0.55); I² = 0%

Test for overall effect: Z = 0.05 (P = 0.96)

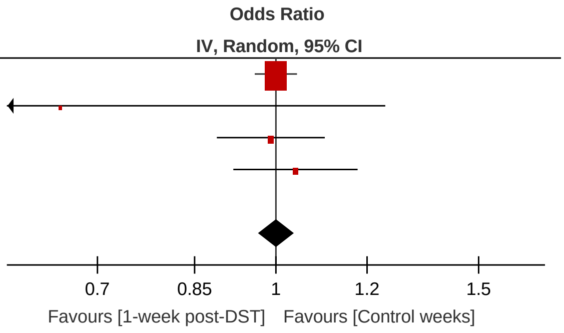

In all studies, "a" = "autumn DST transition", "s" = "spring DST transition".

Figure S14. Risk of acute myocardial infarction (AMI) during the first week following daylight saving time (DST) autumn transition versus control weeks – **Age <65 years only**.

Study or Subgroup	log[Odds Ratio]	SE	Weight	Odds Ratio IV, Random, 95% CI	Year
Janszky 2012a	0	0.0262	88.2%	1.00 [0.95, 1.05]	2012
Kirchberger 2015a	0.0677	0.0715	11.8%	1.07 [0.93, 1.23]	2015
**Total (95% CI)**			**100.0%**	**1.01 [0.96, 1.06]**	

Heterogeneity: Tau² = 0.00; Chi² = 0.79, df = 1 (P = 0.37); I² = 0%

Test for overall effect: Z = 0.33 (P = 0.74)

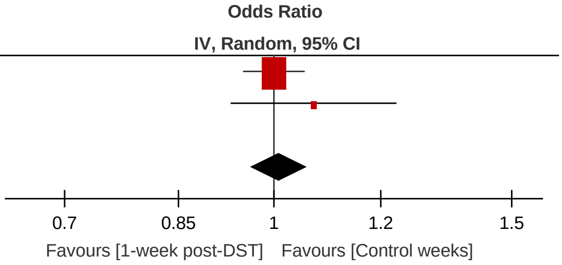

In all studies, "a" = "autumn DST transition", "s" = "spring DST transition".

Figure S15. Risk of acute myocardial infarction (AMI) during the first week following daylight saving time (DST) autumn transition versus control weeks – **Age ≥65 years only**.

Study or Subgroup	log[Odds Ratio]	SE	Weight	Odds Ratio IV, Random, 95% CI	Year	Odds Ratio IV, Random, 95% CI
Janszky 2012a	-0.0101	0.0157	96.2%	0.99 [0.96, 1.02]	2012	
Kirchberger 2015a	-0.0202	0.0786	3.8%	0.98 [0.84, 1.14]	2015	
**Total (95% CI)**			**100.0%**	**0.99 [0.96, 1.02]**		

Heterogeneity: Tau² = 0.00; Chi² = 0.02, df = 1 (P = 0.90); I² = 0%

Test for overall effect: Z = 0.68 (P = 0.50)

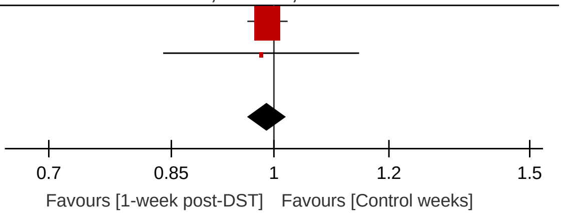

In all studies, "a" = "autumn DST transition", "s" = "spring DST transition".

Figure S16. Funnel plot of the logarithm of the odds ratios vs their standard errors (outcome: risk of AMI during the first week following daylight saving time (DST) transitions versus control weeks – **Overall**).

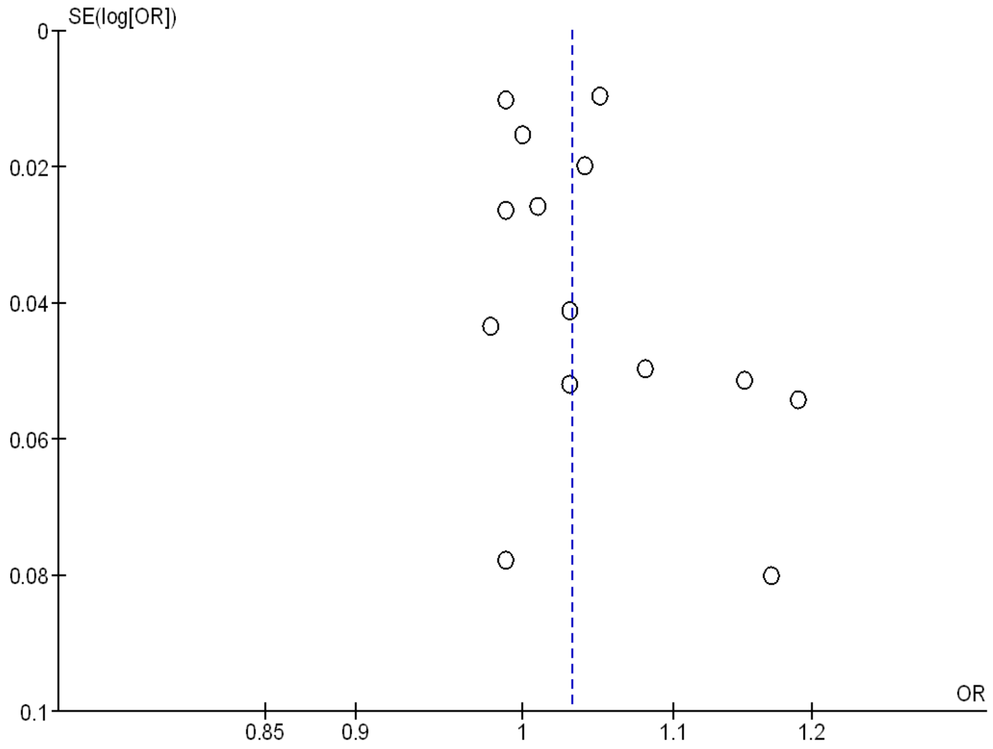

Egger: bias = 0.89 (95% CI = -0.798395 to 2.579988)   P = 0.2729

References

1. Foerch, C.; Korf, H.W.; Steinmetz, H.; Sitzer, M. Abrupt shift of the pattern of diurnal variation in stroke onset with daylight saving time transitions. *Circulation* **2008**, *118*, 284-290.
2. Sipila, J.O.; Ruuskanen, J.O.; Rautava, P.; Kyto, V. Changes in ischemic stroke occurrence following daylight saving time transitions. *Sleep Med* **2016**, *27-28*, 20-24.
3. Lindenberger, L.M.; Ackermann, H.; Parzeller, M. The controversial debate about daylight saving time (DST)-results of a retrospective forensic autopsy study in Frankfurt/Main (Germany) over 10 years (2006-2015). *Int J Legal Med* **2018**, [E-pub ahead of print].
4. Manfredini, R.; Fabbian, F.; Cappadona, R.; Modesti, P.A. Daylight saving time, circadian rhythms, and cardiovascular health. *Intern Emerg Med* **2018**, *13*, 641-646.
